# Restraint of Fumarate Accrual by HIF-1α Preserves miR-27a-Mediated Limitation of Interleukin 10 during Infection of Macrophages by Histoplasma capsulatum

**DOI:** 10.1128/mBio.02710-21

**Published:** 2021-11-09

**Authors:** Heather M. Evans, Destiny F. Schultz, Adam J. Boiman, Melanie C. McKell, Joseph E. Qualls, George S. Deepe

**Affiliations:** a Division of Infectious Diseases, University of Cincinnati College of Medicine, Cincinnati, Ohio, USA; b Immunology Graduate Program, University of Cincinnati College of Medicine, Cincinnati, Ohio, USA; c Department of Pediatrics, University of Cincinnati College of Medicine, Cincinnati, Ohio, USA; d Division of Infectious Diseases, Cincinnati Children's Hospital Medical Center, Cincinnati, Ohio, USA; Texas Christian University

**Keywords:** *Histoplasma*, hypoxia inducible factor 1, lung, innate immunity, mitochondrial metabolism

## Abstract

Hypoxia-inducible factor 1α (HIF-1α) regulates the immunometabolic phenotype of macrophages, including the orchestration of inflammatory and antimicrobial processes. Macrophages deficient in HIF-1α produce excessive quantities of the anti-inflammatory cytokine interleukin 10 (IL-10) during infection with the intracellular fungal pathogen Histoplasma capsulatum (R. A. Fecher, M. C. Horwath, D. Friedrich, J. Rupp, G. S. Deepe, J Immunol 197:565–579, 2016, https://doi.org/10.4049/jimmunol.1600342). Thus, the macrophage fails to become activated in response to proinflammatory cytokines and remains the intracellular niche of the pathogen. Here, we identify the tricarboxylic acid (TCA) cycle metabolite fumarate as the driver of IL-10 during macrophage infection with H. capsulatum in the absence of HIF-1α. Accumulation of fumarate reduced expression of a HIF-1α-dependent microRNA (miRNA), miR-27a, known to mediate decay of *Il10* mRNA. Inhibition of fumarate accrual *in vivo* limited IL-10 and fungal growth. Our data demonstrate the critical role of HIF-1α in shaping appropriate TCA cycle activity in response to infection and highlight the consequences of a dysregulated immunometabolic response.

## INTRODUCTION

Detection of microbial compounds by pattern recognition receptors initiates complex regulatory cascades that regulate global shifts in the metabolic phenotype of innate immune cells. In addition to providing bioenergetic substrates for antimicrobial processes, metabolic by-products serve as signal molecules that instruct the mobilization and resolution of innate immune responses. Recent examples demonstrate this auxiliary role of certain tricarboxylic acid (TCA) cycle metabolites (1). Within macrophages, stimulation with the bacterial ligand lipopolysaccharide (LPS) promotes cytosolic accumulation of citrate, which serves as a cofactor for the acetylation or malonylation of regulators of cytokine production (2–5). LPS stimulation has also been shown to trigger accumulation of succinate, promoting succinylation of protein lysine residues (6–8). Additionally, succinate and fumarate disrupt epigenetic remodeling of histones and DNA via competitive inhibition of α-ketoglutarate-dependent dioxygenases (9, 10).

Hypoxia-inducible factor 1α (HIF-1α) has emerged as an essential regulator of the transition of immune cells into an inflammatory state. While first identified for its role in the response to hypoxia, HIF-1α is a transcription factor that regulates over 1,000 gene targets involved in diverse processes that include cell development, cell-cell interactions, and metabolism. Under normoxic conditions, HIF-1α protein accrues within macrophages stimulated with LPS or infected with a diverse range of microorganisms, including the intracellular fungal pathogen Histoplasma capsulatum (11–14). Within the macrophage, HIF-1α shapes the metabolic shifts required for activation in response to infection and the associated inflammatory and antimicrobial responses (6, 13, 15–21).

H. capsulatum and related *Histoplasma* species are endemic to river valleys around the globe, including those of the central and eastern United States, Latin America, Africa, and Asia, with exposure rates in areas of endemicity reaching as high as 80% (22, 23). Within the host, the pathogen converts to the yeast form and resides in monocytes and macrophages (24). Immunocompetent hosts generally resolve the infection either asymptomatically or with only mild symptoms, provided that the inoculum of H. capsulatum spores does not overwhelm the lung innate immune response. However, in the absence of proinflammatory cues, especially cytokines and activation of T helper responses, infected monocytes and macrophages fail to kill the intracellular yeasts. Unchecked fungal growth within the lungs leads to flu-like illness or, in severe cases, acute respiratory distress syndrome and the development of systemic symptoms as the fungus spreads to additional organ systems (25).

We have previously reported that mice lacking expression of HIF-1α in myeloid cells (*Lyz2cre Hif1α^fl/fl^*) manifest an elevated fungal burden beginning as early as day 3 of infection and succumb to a sublethal inoculum of H. capsulatum (12). The failure of myeloid HIF-1α-deficient hosts to control H. capsulatum growth resulted from exaggerated generation of the cytokine interleukin 10 (IL-10), principally by inflammatory monocytes and macrophages. As a consequence of excessive production of this anti-inflammatory mediator, macrophage responsiveness to gamma interferon (IFN-γ), which activates these cells to exert antifungal activity, was blunted (12).

Our finding of excessive IL-10 production by HIF-1α-deficient monocytes and macrophages is paradoxical because this transcription factor drives IL-10 production (26). To explore how the absence of HIF-1α causes dysregulation of this cytokine, we conducted a series of experiments to unearth the mechanisms underpinning the enhanced synthesis of IL-10. Here, we report a novel mechanism by which HIF-1α inhibits excessive IL-10 production by macrophages during infection with the intracellular fungal pathogen H. capsulatum by preserving a contextually appropriate TCA cycle phenotype. In the absence of restraint by HIF-1α, H. capsulatum infection triggers accumulation of the TCA cycle intermediate fumarate, leading to decreased expression of the microRNA (miRNA) miR-27a in the host macrophage. This relief of miRNA-mediated decay of the *Il10* transcript permits elevated production of IL-10 protein, which promotes unrestrained fungal growth and subsequently a high host mortality.

## RESULTS

### MiR-27a regulates the decay of *Il10* mRNA during H. capsulatum infection.

**FIG 1:**
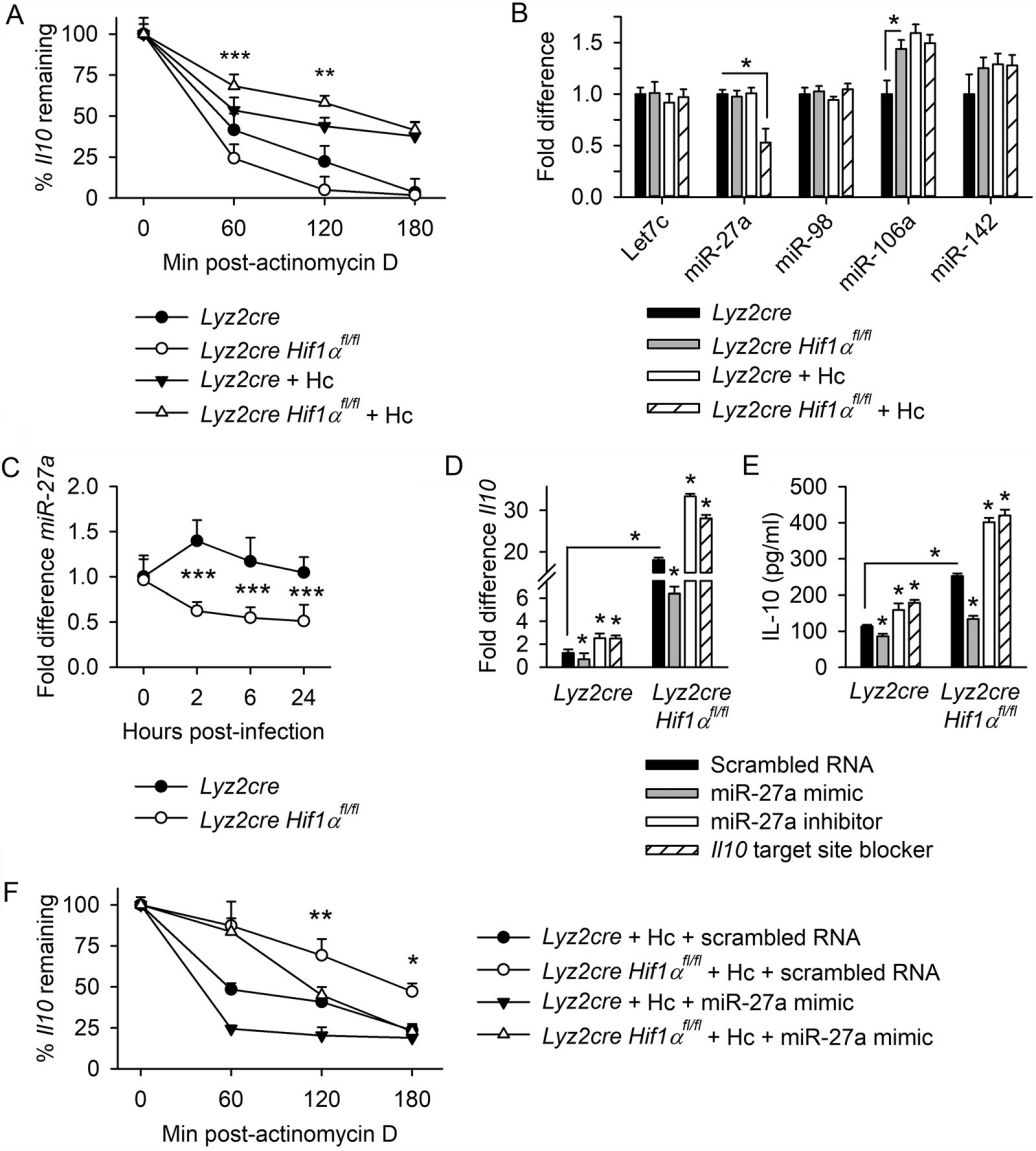
MiR-27a regulates the decay of *Il10* during Hc infection. (A) *Il10* mRNA decay curve, measured by reverse transcription-qPCR (RT-qPCR) at 0 to 180 min of cessation of transcription via actinomycin D treatment in *Lyz2cre* and *Lyz2cre HIF1α^fl/fl^* BMDMs at 24 h postinfection. Data are normalized to the quantity of *Il10* in samples isolated at 0 min. (B) Expression of HIF-1α-dependent miRNAs, quantified by RT-qPCR, in BMDMs at 24 h postinfection. Data are normalized to uninfected controls. (C) miR-27a expression measured by RT-qPCR in BMDMs infected with H. capsulatum (Hc). Data are normalized to uninfected controls. *Il10* mRNA (D) was measured by RT-qPCR at 24 h postinfection, and IL-10 protein (E) was quantified by ELISA at 48 h postinfection in BMDMs previously transfected with a miR-27a mimic (agomir), miR-27a inhibitor (antagomir), or *Il10* 3′ UTR target site blocker. IL-10 protein was below the level of detection in uninfected BMDMs (data not shown). (F) *Il10* mRNA was quantified by RT-qPCR at 0 to 180 min of cessation of transcription via actinomycin D treatment in BMDMs at 24 h postinfection. BMDMs were transfected with scrambled RNA control or miR-27a mimic 18 h prior to infection. Data are normalized to the quantity of *Il10* in samples isolated at 0 min. All data are means and standard errors of the means (SEM). In panels A to D and F, data are from 3 biologically independent samples, representative of 3 experiments. In panel E, data are from 5 biologically independent samples, representative of 3 experiments. For panels B to E, two-way ANOVA with Student-Newman-Keuls *post hoc* testing was used to compare data between the groups. *, *P* < 0.05; ***, *P* < 0.001. For the *Il10* mRNA decay curves, two-way ANOVA with Student-Newman-Keuls *post hoc* testing was used to compare data between the infected *Lyz2cre* and *Lyz2cre HIF1α^fl/fl^* BMDMs (A) or to compare data between the scrambled RNA and mimic-treated *Lyz2cre HIF1α^fl/fl^* BMDMs (F). *, *P* < 0.05; **, *P* < 0.01; ***, *P* < 0.001.

In our model, both HIF-1α-sufficient and -deficient macrophages display elevated quantities of *Il10* mRNA during early infection (12). The quantity of *Il10* mRNA begins to decrease in both groups by 24 h postinfection (hpi), with a more dramatic decrease in *Il10* in the presence of HIF-1α (12). One possible interpretation of these data is that the decay of *Il10* mRNA during infection may be delayed in the absence of HIF-1α, leading to increased translation of *Il10* and, thus, elevated production of IL-10 protein. To evaluate the role of posttranscriptional RNA decay on the production of IL-10, the transcriptional inhibitor actinomycin D was used to measure stability of *Il10* in HIF-1α-sufficient and -deficient bone marrow-derived macrophages (BMDMs) (Fig. 1A). While infection with H. capsulatum increased the half-life (*t*_1/2_) of the *Il10* transcript in both wild-type and HIF-1α-deficient BMDMs, *Il10* mRNA stability was greater in the absence of HIF-1α (*Lyz2cre t*_1/2_ = 124.5 min versus *Lyz2cre Hif1α^fl/fl^ t*_1/2_ = 202.4 min).

Several HIF-1α-dependent miRNAs that target *Il10* have been reported, including miR-27a and members of the Let-7 family (27). Our survey of a panel of relevant miRNAs indicated that infection with H. capsulatum selectively reduced miR-27a expression in HIF-1α-deficient BMDMs (Fig. 1B). No differences in expression of other miRNAs known to regulate *Il10* (specifically, Let-7c, miR-106a, miR-142, and miR-98) were detected between infected HIF-1α-sufficient and -deficient macrophages. miR-106a expression was upregulated in the absence of HIF-1α compared to wild-type control prior to infection, but expression of this miRNA was similar between HIF-1α-sufficient and -deficient cells after infection. Downregulation of miR-27a expression occurred as early as 2 hpi (Fig. 1C).

miR-27a directly targets the 3′ untranslated region (UTR) of the *Il10* transcript, and downregulation of miR-27a indirectly increases IL-10 protein and tempers proinflammatory cytokine production in Toll-like receptor 2 (TLR2)- and TLR4-stimulated macrophages (27). To evaluate the impact of miR-27a activity on *Il10* mRNA and protein expression, BMDMs were transfected with a 30 nM concentration of a miR-27a mimic (agomir) or a miR-27a inhibitor (antagomir) for 18 h prior to infection (Fig. 1D and E). Treatment with a miR-27a mimic restrained the quantity of *Il10* mRNA detected at 24 h postinfection and IL-10 protein at 48 h postinfection in HIF-1α-deficient BMDMs compared to cells transfected with scrambled RNA, while transfection with miR-27a inhibitor boosted the quantity of *Il10* mRNA and IL-10 protein in both HIF-1α-sufficient and -deficient BMDMs (Fig. 1D and E). miRNA mimics and inhibitors broadly regulate the full spectrum of activity for the miRNA of interest and thus lack specificity. To directly explore the role of activity at the 3′ UTR of the *Il10* transcript in the stability of *Il10* and production of IL-10, cells were transfected with a small RNA target site blocker designed to protect the known binding site of miR-27a at the 3′ UTR of *Il10* (27). The presence of this target site blocker increased the quantity of *Il10* mRNA and IL-10 protein in both HIF-1α-sufficient and -deficient BMDMs compared to cells transfected with scrambled RNA (Fig. 1D and E). As a control for specificity, we examined the effect of these alterations on an irrelevant gene, *Tnfα*. Transfection with miR-27a mimic, inhibitor, or the target site blocker did not alter expression of this cytokine (Fig. S1). Transfection with miR-27a mimic decreased the half-life of *Il10* by 53.0% and 42.2% in wild-type and HIF-1α-deficient cells, respectively (Fig. 1F).

10.1128/mBio.02710-21.1 FIG S1 Modulation of miR-27a activity does not alter TNF-α expression. TNF-α protein was quantified by ELISA at 48 h postinfection in BMDMs previously transfected with a miR-27a mimic (agomir), miR-27a inhibitor (antagomir), or *Il10* 3′ UTR target site blocker. Data are means and SEM for 5 biologically independent samples, representative of 3 experiments. Two-way ANOVA with Student-Newman-Keuls *post hoc* testing was used to compare data between groups. ns, not significant (*P* > 0.05). Download FIG S1, TIF file, 2.2 MB. Copyright © 2021 Evans et al. 2021 Evans et al. https://creativecommons.org/licenses/by/4.0/ This content is distributed under the terms of the Creative Commons Attribution 4.0 International license.

### The absence of HIF-1α promotes elevated TCA cycle activity and mitochondrial respiration.

H. capsulatum infection induces accrual of HIF-1α (12). Thus, the expression of miR-27a prior to infection occurs independently of regulation by HIF-1α. During infection, HIF-1α is required to maintain basal expression of miR-27a (Fig. 1B and C). In the absence of this transcription factor, infection with H. capsulatum is accompanied by reduced expression of miR-27a. This pattern of expression suggests a two-hit model, in which HIF-1α-deficiency and the stimulus of infection are both required for differential expression of miR-27a. Dramatic shifts in cellular metabolism are critical determinants of the ability or failure of a macrophage to respond appropriately to infection (18, 19). In this context, HIF-1α-regulated metabolites serve as both fuel and signal for proinflammatory and antimicrobial responses (6). To explore the role of HIF-1α-mediated metabolic changes in the regulation of miR-27a and IL-10, we first sought to conduct a broad analysis of the metabolic phenotype of HIF-1α-sufficient and -deficient cells before and after infection with H. capsulatum.

**FIG 2:**
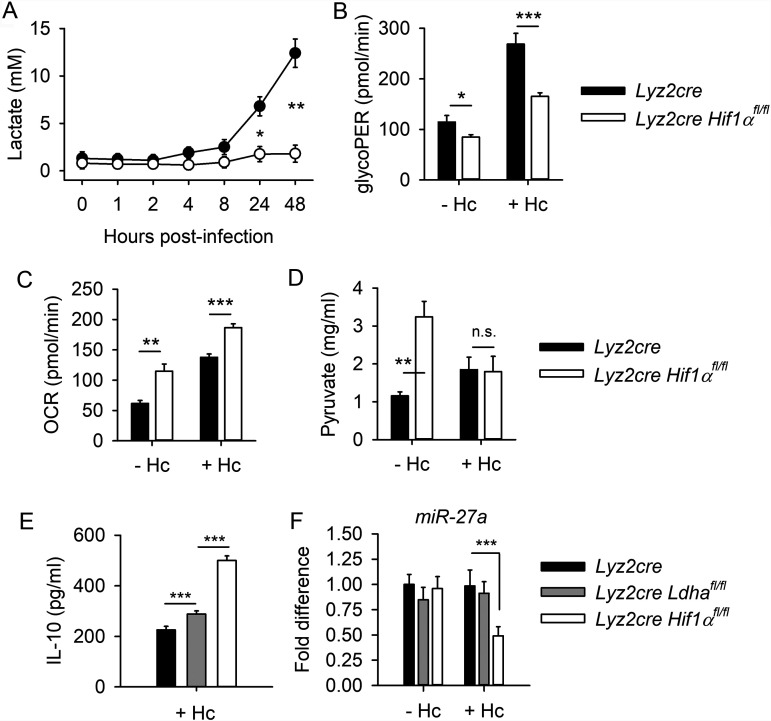
HIF-1α regulates the metabolism of Hc-infected BMDMs. (A) Serial measurements of extracellular lactate were quantified with a commercial kit. Basal glycolysis (glycolytic proton efflux rate [glycoPER]) (B) and basal mitochondrial respiration (oxygen consumption rate [OCR]) (C) were measured using the Seahorse XFe96 analyzer (Agilent). (D) Intracellular pyruvate quantity was measured with a commercial kit. (E) Extracellular IL-10 protein production by BMDMs was measured by ELISA at 48 hpi. (F) miR-27a expression by BMDMs was measured by qPCR at 27 hpi. (A to F) Data are means and SEM and are representative of 2 separate experiments. Data in panels A and F are from 4 biologically independent samples; data in panels B to E are from 6 biologically independent samples. For all panels, two-way ANOVA with Student-Newman-Keuls *post hoc* testing was used to compare data between groups. *, *P* < 0.05; **, *P* < 0.01; ***, *P* < 0.001; n.s., not significant.

Recent studies from our group indicate that H. capsulatum infection increases both glycolysis (as measured by the basal extracellular acidification rate [ECAR]) and mitochondrial respiration in human monocyte-derived macrophages and in mouse BMDMs (28). Conversely, BMDMs deficient in HIF-1α display a preference for elevated mitochondrial respiration during infection, with a limited increase in ECAR compared to the higher ECAR of HIF-1α-expressing controls (28). Here, we expanded these studies to more fully illustrate the metabolic profile in the absence of HIF-1α. To confirm the regulation of glycolysis by HIF-1α in macrophages infected with H. capsulatum, serial extracellular measurements of lactate were analyzed by colorimetric assay (Fig. 2A). HIF-1α-deficient BMDMs secreted less lactate than wild-type controls by 24 hpi. Likewise, the glycolytic rate assay revealed that the basal glycolytic proton efflux rate (glycoPER) is reduced in the absence of HIF-1α in both infected and uninfected BMDMs compared to wild-type controls (Fig. 2B).

The mitochondrial stress assay indicated that basal mitochondrial respiration is elevated in the absence of HIF-1α in both infected and uninfected BMDMs compared to controls (Fig. 2C). While lactate production was deficient in the absence of HIF-1α, intracellular pyruvate was higher in HIF-1α-deficient BMDMs compared to controls prior to infection (Fig. 2D). During infection, quantities of pyruvate were similar between the groups (Fig. 2D). Other measurements of glycolytic activity did not reveal a defect in HIF-1α-deficient cells. Specifically, HIF-1α-deficient BMDMs did not display diminished glucose transporter 1 (Glut1) surface expression, 2-[*N*-(7-nitrobenz-2-oxa-1,3-diazol-4-yl)amino]-2-deoxy-d-glucose (2-NBDG) uptake, hexokinase activity, or glucose-6-phosphate dehydrogenase (G6DPH) activity compared to HIF-1α-sufficient controls (Fig. S2A to D).

10.1128/mBio.02710-21.2 FIG S2 The absence of HIF-1α does not reduce early glucose uptake or hexokinase activity. (A) Expression of glucose transporter (Glut1) on infected BMDMs was measured by flow cytometry. (B) Relative glucose uptake was measured via incubation of BMDMs with the fluorescent d-glucose analog 2-NBDG. Hexokinase activity (C) and glucose-6-phosphate dehydrogenase (G6PDH) activity (D) were quantified by colorimetric assay at 24 h postinfection. (A to D) Data are means and SEM for 5 biologically independent samples, representative of 2 experiments. Two-way ANOVA with Student-Newman-Keuls *post hoc* testing was used to compare data between groups; *, *P* < 0.05. Download FIG S2, TIF file, 0.2 MB. Copyright © 2021 Evans et al. 2021 Evans et al. https://creativecommons.org/licenses/by/4.0/ This content is distributed under the terms of the Creative Commons Attribution 4.0 International license.

The limited lactate production and ECAR of HIF-1α deficient BMDMs is unsurprising, as the gene encoding lactate dehydrogenase (*Ldha*) is a known target of HIF-1α (29). To evaluate the role of decreased lactate production in H. capsulatum infection, BMDMs deficient in *Ldha* expression were generated from *Lyz2cre Ldha^fl/fl^* mice. *Ldha*-deficient BMDMs produced slightly elevated IL-10 levels compared to *Lyz2cre* controls during infection (Fig. 2E) without altered miR-27a expression (Fig. 2F). These data indicate that decreased Ldha activity is not the primary driver of excessive IL-10 in HIF-1α-deficient BMDMs.

### A “broken” TCA cycle drives accumulation of fumarate in the absence of HIF-1α.

**FIG 3:**
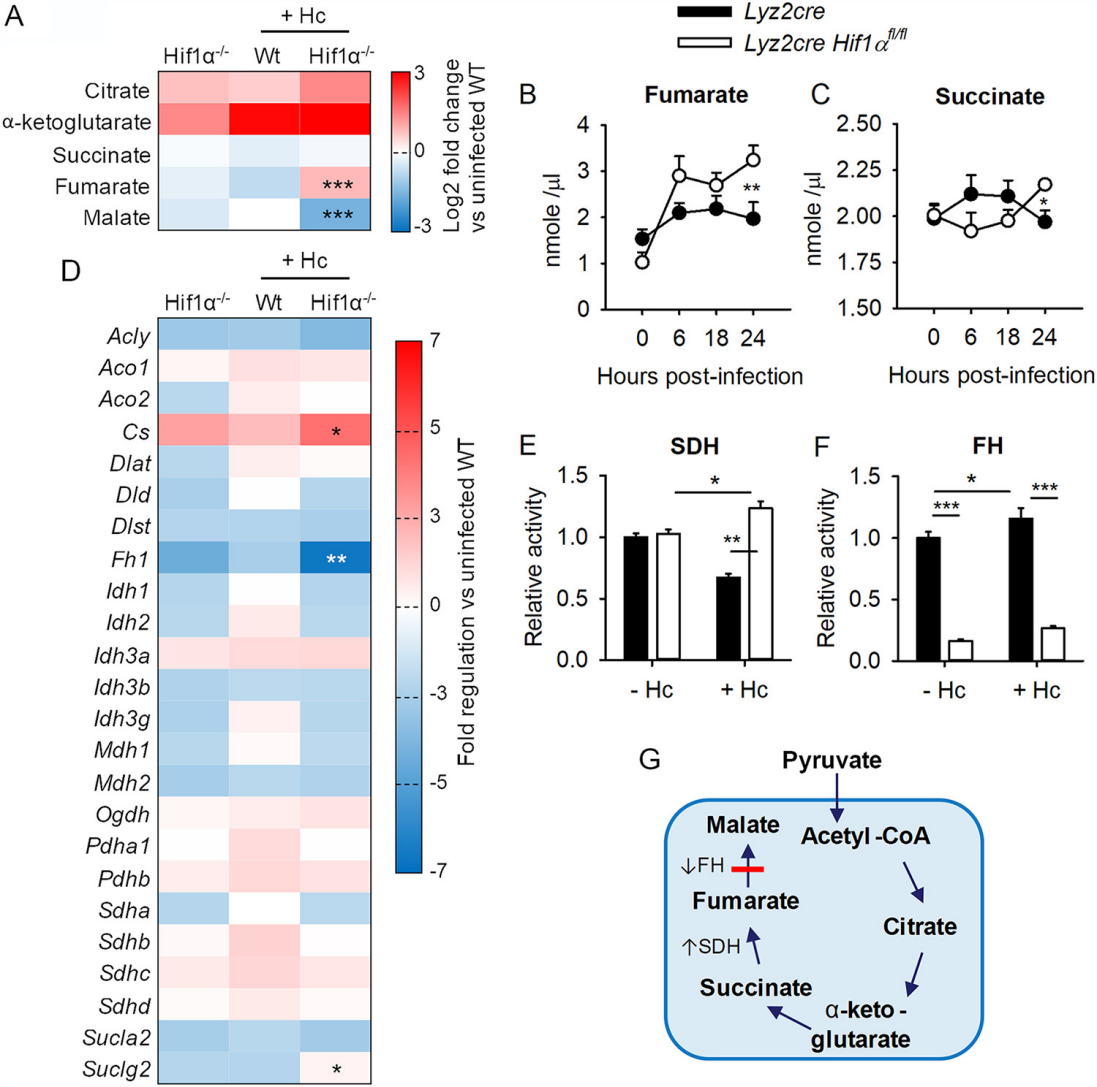
TCA cycle activity is elevated in macrophages during Hc infection in the absence of HIF-1α. (A) TCA cycle metabolites were quantified by LC-MS at 24 h postinfection. Data are pooled from 3 separate experiments and represent the mean log_2_ fold change versus uninfected *Lyz2cre* BMDMs from 4 biologically independent samples per experiment. Serial measurements of intracellular fumarate (B) and succinate (C) were quantified by commercial kit and are presented as means and SEM from 4 biologically independent samples (representative of 2 experiments). (D) Expression of TCA cycle enzyme mRNAs was quantified by glycolysis RT^2^ Profiler PCR array (Qiagen); data were pooled from 3 separate experiments with 1 biologically independent sample per experiment. (E and F) Intracellular activities of SDH and FH were quantified by microplate activity assay. Data are means and SEM from 4 biologically independent samples (representative of 2 experiments). (A and D) Two-tailed Student’s *t* tests were used to compare data between infected *Lyz2cre* and *Lyz2cre Hif1α^fl/fl^* groups. *, *P* < 0.05; **, *P* < 0.01; ***, *P* < 0.001. (B, C, E, and F) Two-way ANOVA with Student-Newman-Keuls *post hoc* testing was used to compare data between groups. *, *P* < 0.05; **, *P* < 0.01; ***, *P* < 0.001. (G) Model of the TCA cycle in infected HIF-1α-deficient macrophages.

While HIF-1α-deficient BMDMs may be deficient in the conversion of pyruvate to lactate, the cells appear to produce sufficient pyruvate to fuel metabolic processes in the mitochondrion (Fig. 2). To further characterize HIF-1α-dependent mitochondrial metabolism during H. capsulatum infection, we quantified intracellular TCA cycle metabolites via liquid chromatography-mass spectrometry (LC-MS) and colorimetric kit assay (Fig. 3A to C). Intracellular quantities of fumarate were higher in HIF-1α-deficient BMDMs compared to wild-type by 24 hpi (Fig. 3A and B). Additionally, infected HIF-1α-deficient macrophages contained slightly more succinate (Fig. 3A and C) but less malate (Fig. 3A) at 24 hpi than HIF-1α-sufficient cells. The expression of a panel of genes related to the TCA cycle was analyzed by quantitative PCR (qPCR). Two of the early TCA cycle genes, *Cs* (which encodes citrate synthase) and *Suclg2* (which encodes succinate coenzyme A [succinate-CoA] ligase subunit beta), were upregulated in infected HIF-1α-deficient BMDMs compared to infected wild-type cells (Fig. 3D). Expression of the gene encoding fumarate hydratase (*Fh1*), which converts fumarate to malate, was downregulated in HIF-1α-deficient BMDMs during infection compared to controls (Fig. 3D). The relative activity of succinate dehydrogenase (SDH), which converts succinate to fumarate, was elevated in infected HIF-1α-deficient cells compared to wild-type controls (Fig. 3E). In contrast, relative fumarate hydratase (FH) activity was decreased in HIF-1α-deficient cells compared to wild-type controls independently of infection (Fig. 3F). These data indicate that HIF-1α maintains appropriate TCA cycle activity during infection with H. capsulatum. Conversely, in the absence of HIF-1α, increased SDH activity paired with a “break” in FH-mediated production of malate promotes accumulation of fumarate. Changes in SDH activity were not attributable to differences in *Sdh* expression but were dependent on both infection and the presence or absence of HIF-1α. Conversely, decreased FH activity in HIF-1α-deficient BMDMs compared to wild-type controls correlated with diminished expression of *Fh* and occurred independently of infection.

### Inhibition of the TCA cycle restrains IL-10 production during H. capsulatum infection.

**FIG 4:**
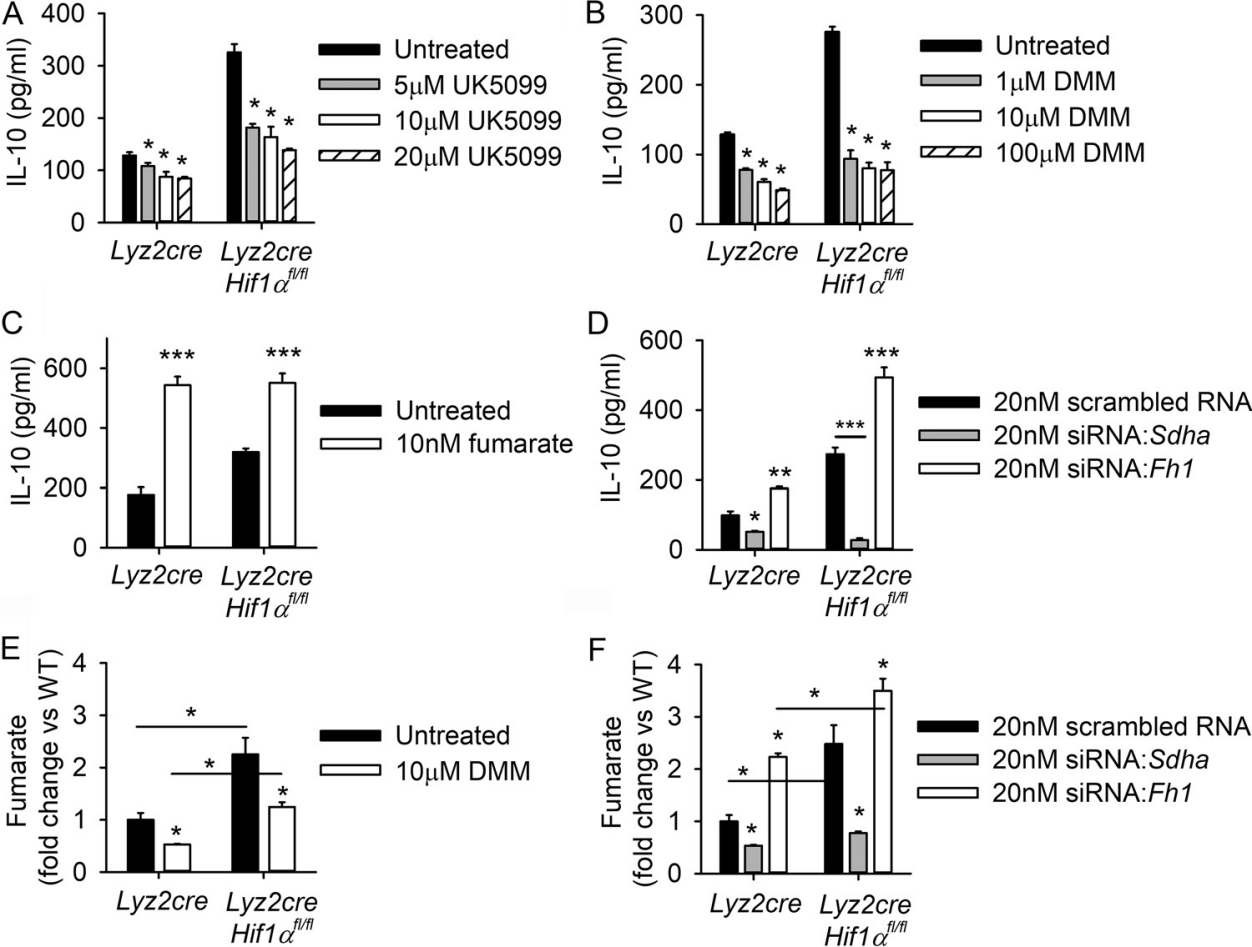
Inhibition of the TCA cycle restrains IL-10 during H. capsulatum infection. (A to C) IL-10 cytokine production was quantified by ELISA at 48 h after concurrent Hc infection and treatment with (A) UK5099, (B) DMM, or (C) dimethyl fumarate. (D) BMDMs were transfected with 20 nM scrambled RNA control, 20 nM siRNA-*Sdha*, or 20 nM siRNA-*Fh1* for 18 h prior to Hc infection. IL-10 was quantified by ELISA at 48 h postinfection. Intracellular fumarate was quantified with a commercial kit at 24 h postinfection in cells treated with DMM (E) or siRNAs (F). (A to D) Data are means and SEM for 4 biologically independent samples per group and are representative of 3 separate experiments. (E and F) Data are mean fold change versus uninfected wild-type control and SEM (*n* = 10), pooled from 2 separate experiments. For all panels, two-way ANOVA with Student-Newman-Keuls *post hoc* testing was used to compare data between groups. *, *P* < 0.05; **, *P* < 0.01; ***, *P* < 0.001.

The anti-inflammatory effects of metabolites have been widely reported; fumarate is associated with increased IL-10 (30–32). To test the association of elevated TCA cycle activity with excessive IL-10 production in the absence of HIF-1α, IL-10 production was quantified by enzyme-linked immunosorbent assay (ELISA) at 48 h after concurrent H. capsulatum infection and treatment with TCA cycle inhibitors (Fig. 4A and B). Treatment with UK5099, an inhibitor of pyruvate transport into the mitochondrion, and dimethyl malonate (DMM), a competitive inhibitor of the conversion of succinate to fumarate by succinate dehydrogenase, reduced IL-10 production in both HIF-1α-sufficient and -deficient infected BMDMs (Fig. 4A and B). The decrease in IL-10 in the absence of HIF-1α was more dramatic, with 10 μM UK5099 leading to a 50% decrease in IL-10 in HIF-1α-deficient cells compared to a 32% decrease in wild-type cells. Treatment with 10 μM DMM produced a 71% decrease in IL-10 in HIF-1α-deficient cells compared to a 53% decrease in wild-type controls (Fig. 4A and B). Inhibition of the TCA cycle with 10 μM UK5099 and DMM did not lead to nonspecific effects such as direct killing of H. capsulatum (Fig. S3A and B), decreased macrophage viability (Fig. S3C and D), decreased production of IL-1β and tumor necrosis factor alpha (TNF-α) (Fig. S3E and F), or suppression of mitochondrial respiration (Fig. S3G). Treatment with exogenous fumarate increased IL-10 (Fig. 4C) but not TNF-α (Fig. S3H) in infected HIF-1α-sufficient and -deficient BMDMs. Exogenous fumarate did not alter mitochondrial respiration (Fig. S3G).

10.1128/mBio.02710-21.3 FIG S3 Inhibition of TCA cycle does not negatively impact H. capsulatum viability or BMDM viability, cytokine production, and mitochondrial respiration. The impact of UK5099 (A) and DMM (B) on the viability of Hc was assessed after 24 h growth in drug-treated medium (complete RPMI) or *Lyz2cre* or *Lyz2cre HIF1α^fl/fl^* BMDMs. Data are presented relative to CFU of H. capsulatum in drug-free medium or BMDMs. The impact of UK5099 (C) and DMM (D) on BMDM viability was assessed by flow cytometry with 7-AAD staining after 48 h in drug-treated medium. Data were normalized to the viability of untreated controls. IL-1β (E) and TNF-α (F) production was evaluated by ELISA after 48 h of concurrent TCA cycle inhibition and Hc infection. (G) The impact of UK5099, DMM, and exogenous fumarate on basal mitochondrial respiration (OCR) was measured by Seahorse XFe96 analyzer. (H) TNF-α production was quantified by ELISA after 48 h of concurrent exogenous fumarate treatment and Hc infection. (I to K) BMDMs were transfected with 20 nM scrambled RNA control, 20 nM siRNA-*Sdha*, or 20 nM siRNA-*Fh1* for 18 h prior to Hc infection. *Sdha* (I), *Fh1* (J), and *Il10* (K) mRNA was measured by RT-qPCR at 24 h postinfection. (A to H) Data are means and SEM for 4 biologically independent samples and are representative of 2 separate experiments. (G to I) Data are means and SEM for 3 biologically independent samples and are representative of 3 experiments. For all panels, two-way ANOVA with Student-Newman-Keuls *post hoc* testing was used to compare data between groups. *, *P* < 0.05; **, *P* < 0.01; ***, *P* < 0.001. Download FIG S3, TIF file, 0.5 MB. Copyright © 2021 Evans et al. 2021 Evans et al. https://creativecommons.org/licenses/by/4.0/ This content is distributed under the terms of the Creative Commons Attribution 4.0 International license.

Collectively, these data implicate fumarate in the production of excessive IL-10 in HIF-1α-deficient BMDMs. To more specifically address the role of fumarate, small interfering RNAs (siRNAs) were employed to knock down expression of *Sdha* and *Fh1* during infection (Fig. S3I to K). Transfection with siRNA targeting *Sdha* prior to infection decreased the quantities of *Il10* mRNA (Fig. S3K) at 24 h postinfection and IL-10 protein at 48 h postinfection (Fig. 4D) in both HIF-1α-sufficient and -deficient BMDMs. Conversely, transfection with siRNA targeting *Fh1* increased the quantities of *Il10* mRNA (Fig. S3K) at 24 h postinfection and IL-10 protein at 48 h postinfection (Fig. 4D) in both HIF-1α-sufficient and -deficient BMDMs.

To confirm the positive association between fumarate and the production of IL-10, the intracellular quantity of this metabolite was assayed following manipulation of the TCA cycle. Inhibition of succinate dehydrogenase with 10 μM DMM decreased intracellular quantities of fumarate in both HIF-1α-sufficient and -deficient BMDMs at 24 h postinfection (Fig. 4E). Silencing of *Sdha* decreased intracellular quantities of fumarate in both HIF-1α-sufficient and -deficient BMDMs at 24 h postinfection, while knockdown of *Fh1* increased the concentration of fumarate (Fig. 4F).

### Fumarate decreases miR-27a expression.

**FIG 5:**
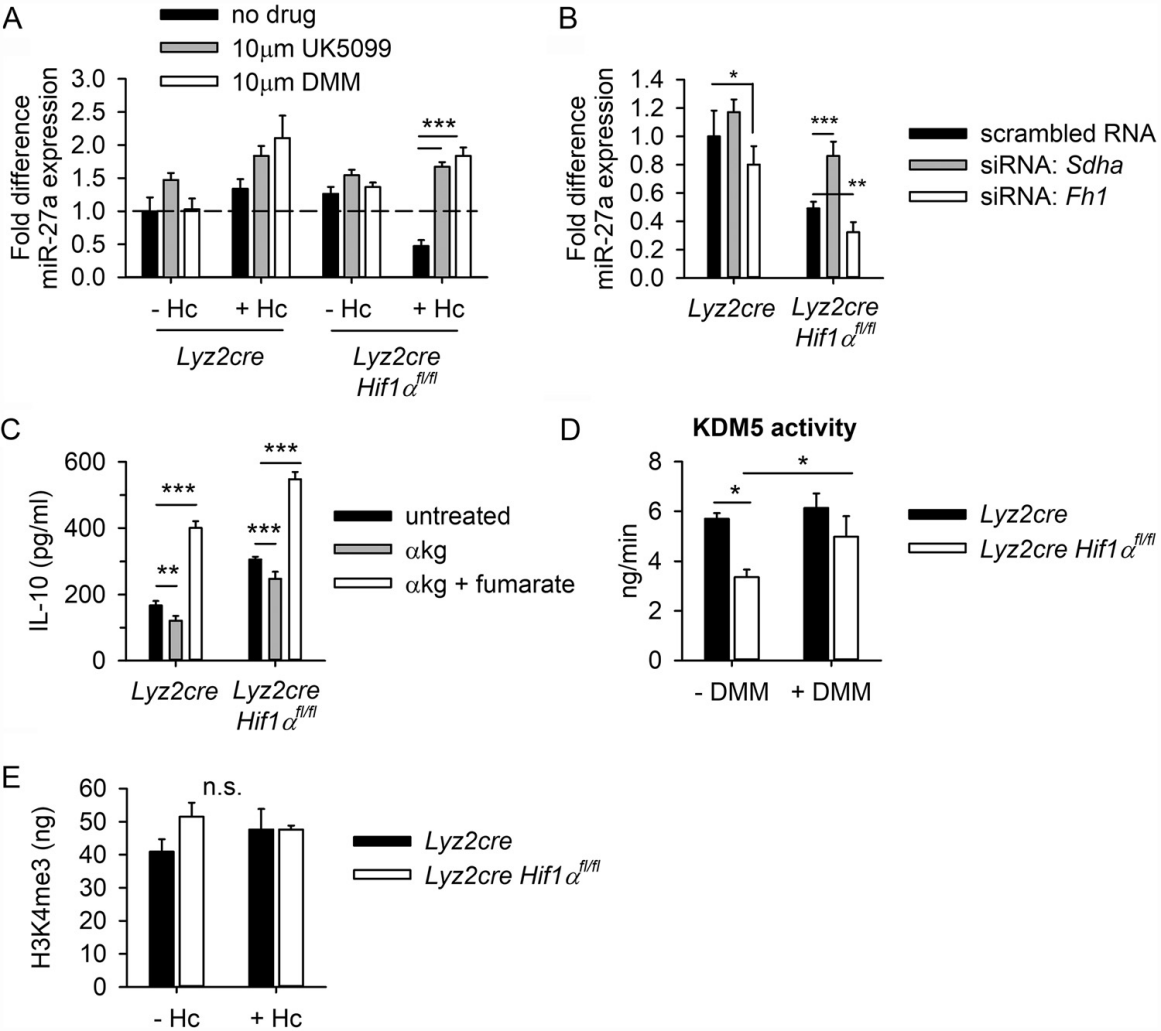
Fumarate decreases miR-27a expression. (A) miR-27a expression was quantified by RT-qPCR at 24 h after concurrent Hc infection and treatment with 10 μM UK5099 or DMM. Data are mean fold difference relative to uninfected *Lyz2cre* control, with SEM, for 4 biologically independent samples, representative of 2 experiments. (B) BMDMs were transfected with 20 nM scrambled RNA control, 20 nM siRNA-*Sdha*, or 20 nM siRNA-*Fh1* for 18 h prior to Hc infection. MiR-27a expression was measured by RT-qPCR at 24 h postinfection. Data are means and SEM for 3 biologically independent samples, representative of 2 experiments. (C) IL-10 protein was quantified by ELISA after 48 h of concurrent Hc infection and treatment with 1 mM exogenous α-ketoglutarate (α-kg) and 10 nM dimethyl fumarate. Data are means and SEM for 5 biologically independent samples, representative of 2 experiments. (D) KDM5 demethylase activity was quantified by fluorometric assay after 24 h of concurrent Hc infection and treatment with 10 μM dimethyl malonate (DMM). (E) H3K4me3 was quantified by ELISA using total protein samples extracted from BMDMs at 24 h postinfection. Data are means and SEM for 7 biologically independent samples, pooled from 2 separate experiments. For all panels, two-way ANOVA with Student-Newman-Keuls *post hoc* testing was used to compare data between groups; *, *P* < 0.05; **, *P* < 0.01; ***, *P* < 0.001.

Our data indicate that in the absence of HIF-1α, infection of macrophages with H. capsulatum leads to decreased expression of miR-27a and, thus, preservation of the *Il10* transcript and increased production of IL-10 protein. To test if elevated TCA cycle activity accounts for reduced miR-27a expression during H. capsulatum infection, we quantified miR-27a in BMDMs at 24 h after concurrent infection and treatment with the TCA cycle inhibitors UK5099 and DMM (Fig. 5A). Inhibition of pyruvate transport into the mitochondrion with UK5099 or competitive inhibition of SDH with DMM elevated miR-27a expression in both HIF-1α-sufficient and -deficient BMDMs (Fig. 5A). siRNA-mediated knockdown of *Sdha* increased miR-27a expression, while knockdown of *Fh1* further decreased miR-27a expression in infected HIF-1α-deficient macrophages (Fig. 5B) These data reveal that accumulation of fumarate in the absence of HIF-1α drives production of IL-10 via downregulation of miR-27a.

### miR-27a expression is correlated with α-ketoglutarate-dependent dioxygenase activity during H. capsulatum infection.

TCA cycle metabolites, including fumarate, exert epigenetic effects (33, 34). Certain DNA and histone dioxygenases use α-ketoglutarate as a cofactor. Due to its structural similarities to α-ketoglutarate, fumarate functions as a competitive inhibitor of α-ketoglutarate-dependent dioxygenases, including JmjC domain-containing histone demethylates (KDMs) and the Tet (10-11 translocation) DNA hydroxylases (10). Fumarate inhibits Tet-mediated demethylation of a CpG island in the promoter region of the miRNA cluster miR-200, suppressing the expression of these antimetastatic miRNAs (35). Loss of Tet2 expression is associated with elevated plasma IL-10 (36). In macrophages, fumarate inhibits KDM5-mediated histone demethylation (9).

To assess the relevance of competitive inhibition of α-ketoglutarate-dependent dioxygenases to control of IL-10, we tested the effect of treatment with exogenous α-ketoglutarate on the expression of IL-10 protein (Fig. 5C). Treatment with 1 mM dimethyl α-ketoglutarate decreased IL-10 protein in HIF-1α-deficient macrophages, while treatment with 10 nM dimethyl fumarate reversed this effect (Fig. 5C). Treatment with exogenous α-ketoglutarate did not lead to detectable changes in intracellular fumarate (Fig. S4). Nuclear extracts collected from infected HIF-1α-deficient BMDMs and controls at 24 h postinfection were assayed for total KDM5 histone demethylase activity (Fig. 5D). Total KDM5 activity was decreased in infected HIF-1α-deficient macrophages compared to wild-type controls (Fig. 5D). Treatment with DMM restored KDM5 activity in HIF-1α-deficient macrophages (Fig. 5D). These data implicate decreased KDM5 histone demethylation activity as the mechanism of fumarate-mediated regulation of miR-27a expression and, thus, *Il10*. The KDM5 subfamily is responsible for removal of trimethylation from the 4th lysine of the histone H3 protein (H3K4me3). Protein extracts from infected HIF-1α-deficient BMDMs and controls were assayed for H3K4me3 at 24 h postinfection (Fig. 5E). No significant differences in total quantity of H3K4me3 were observed between HIF-1α-deficient BMDMs and controls before or after infection. This evidence suggests that the changes in total KDM5 activity observed in Fig. 5D may be insufficient to alter H3K4me3 at a time point relevant for regulation of miR-27a expression.

10.1128/mBio.02710-21.4 FIG S4 Treatment with exogenous α-ketoglutarate does not alter intracellular fumarate. Intracellular fumarate was quantified by commercial kit after 24 h of concurrent Hc infection and treatment with 1 mM exogenous α-ketoglutarate (α-kg). Data are mean fold change versus uninfected, untreated wild-type control and SEM (*n* = 10), pooled from 2 separate experiments. Two-way ANOVA with Student-Newman-Keuls *post hoc* testing was used to compare data between groups; *, *P* < 0.05; **, *P* < 0.01; ***, *P* < 0.001. Download FIG S4, TIF file, 1.9 MB. Copyright © 2021 Evans et al. 2021 Evans et al. https://creativecommons.org/licenses/by/4.0/ This content is distributed under the terms of the Creative Commons Attribution 4.0 International license.

### Fumarate drives IL-10 and increased fungal burden *in vivo*.

**FIG 6:**
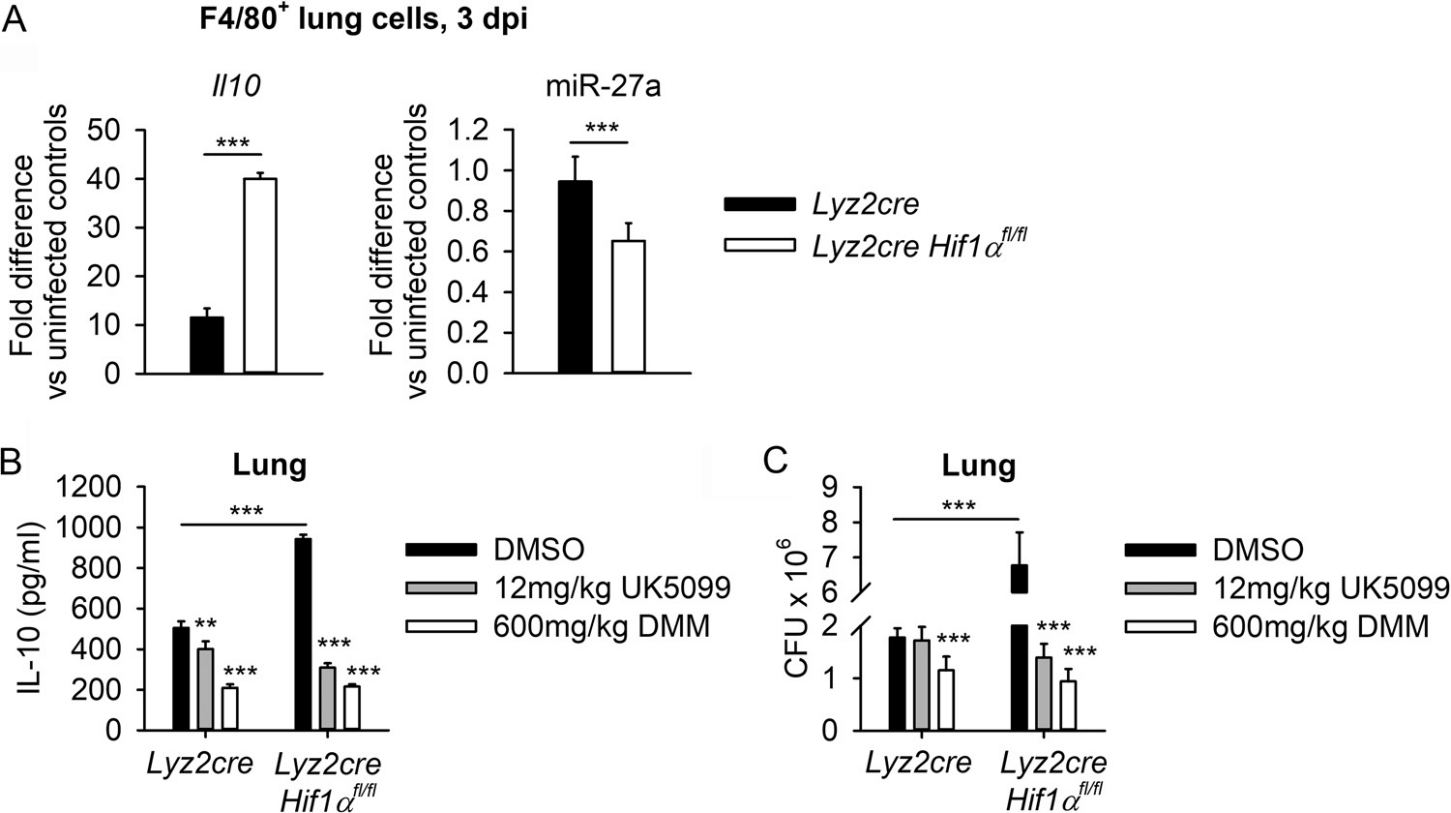
TCA cycle activity drives IL-10 and increased fungal burden *in vivo*. (A) F4/80^+^ cells were sorted from lung homogenate at day 3 postinfection in *Lyz2cre* and *Lyz2cre Hif1α^fl/fl^* mice. *Il10* mRNA and miR-27a were measured by RT-qPCR, with data normalized to expression in uninfected F4/80^+^ lung cells. Data are means and SEM for 6 biologically independent samples, pooled from 2 experiments. (B and C) Lungs were collected from *Lyz2cre* and *Lyz2cre Hif1α^fl/fl^* mice at day 3 postinfection, after 4 days of treatment with DMSO (vehicle), 12 mg/kg UK5099, or 600 mg/kg DMM. (B) Lung IL-10, quantified by ELISA. (C) Lung fungal burden, quantified by CFU. Data are means and SEM for 6 biologically independent samples, pooled from 2 experiments. For all panels, two-way ANOVA with Student-Newman-Keuls *post hoc* testing was used to compare data between the groups. **, *P* < 0.01; ***, *P* < 0.001.

To confirm the negative correlation between miR-27a and *Il10* in a mouse model of histoplasmosis, F4/80^+^ macrophages were isolated from the lungs of *Lyz2cre Hif1α^fl/fl^* mice at day 3 postinfection (Fig. 6A). We chose 3 days because we had previously demonstrated an increased fungal lung burden on day 3 in *Lyz2cre Hif1α^fl/fl^* mice versus wild-type controls (12). The decreased expression of miR-27a and increased quantity of *Il10* mRNA observed in *Lyz2cre Hif1α^fl/fl^* mice versus wild-type controls (Fig. 6A) was comparable to that observed in BMDMs (Fig. 1A).

As a proof-of-concept experiment to evaluate the effects of TCA cycle inhibition *in vivo*, *Lyz2cre* and *Lyz2cre Hif1α^fl/fl^* mice were pretreated with 12 mg/kg UK5099, 600 mg/kg DMM, or vehicle (dimethyl sulfoxide [DMSO]) 1 day prior to Hc infection and daily until termination at day 3 postinfection. Inhibitor doses were chosen based on previous use in mice by other groups (37, 38). Cessation of the experiment at day 3 postinfection provided an early time point known to yield differential fungal lung burdens in *Lyz2cre* and *Lyz2cre Hif1α^fl/fl^* mice (12), while minimizing off-target effects of TCA cycle inhibition on the adaptive immune response. UK5099 has been shown to inhibit activation of CD4^+^ T cells (39). HIF-1α preserves the capacity of the macrophage to respond to IFN-γ and thus control H. capsulatum infection (12). While CD4^+^ and CD8^+^ T cells are the predominate source of IFN-γ later in infection, αβ T-cell receptor-negative (TCR^−^) cells (presumably γδ T cells, natural killer cells, or professional antigen-presenting cells) produce modest quantities of lung IFN-γ as early as day 3 postinfection (40, 41). Here, treatment with UK5099 and DMM limited IL-10 production in the lungs (Fig. 6B) in both *Lyz2cre* and *Lyz2cre Hif1α^fl/fl^* mice and limited the fungal lung burden (Fig. 6C) in *Lyz2cre Hif1α^fl/fl^* mice. As a control, we asked if these compounds altered production of TNF-α. *In vivo* treatment with UK5099 and DMM had no effect on total lung TNF-α at 3 dpi (Fig. S5). These data suggest that limitation of fumarate accumulation enhances host control of H. capsulatum infection.

10.1128/mBio.02710-21.5 FIG S5 Inhibition of the TCA cycle *in vivo* does not negatively affect TNF-α production. TNF-α was quantified by ELISA from the homogenate of lungs collected from *Lyz2cre* and *Lyz2cre Hif1α^fl/fl^* mice at day 3 postinfection, after 4 days of treatment with DMSO (vehicle), 12 mg/kg UK5099, or 600 mg/kg DMM. Data are means and SEM for a total of 7 biologically independent samples pooled from two experiments. Download FIG S5, TIF file, 2.2 MB. Copyright © 2021 Evans et al. 2021 Evans et al. https://creativecommons.org/licenses/by/4.0/ This content is distributed under the terms of the Creative Commons Attribution 4.0 International license.

## DISCUSSION

Our previous report demonstrated that excessive IL-10 production by HIF-1α-deficient myeloid-derived macrophages during intracellular infection with the fungal pathogen H. capsulatum inhibits the capacity of the macrophage to respond to proinflammatory cytokines, especially IFN-γ from T cells, resulting in unchecked fungal growth and host death (12). In this study, we demonstrated that the transcription factor HIF-1α preserves the capacity of myeloid-derived macrophages to control fungal growth by maintaining basal expression of a known negative regulator of *Il10* stability, miR-27a. In the absence of HIF-1α, accumulation of the TCA cycle intermediate fumarate leads to decreased expression of miR-27a and, thus, preservation of the *Il10* transcript. Here, we demonstrate that disruption of the TCA cycle via inhibition of SDH activity or blockage of pyruvate transport into the mitochondrion is sufficient to preserve basal miR-27a expression, restrain IL-10 production, and restore the capacity of the host to control fungal growth even in the absence of HIF-1α. These data reveal a novel HIF-1α/fumarate/miR-27a axis that highlights the significance of TCA cycle metabolism in shaping appropriate macrophage responses to infection.

HIF-1α is known to promote the expression of the IL-10-regulating miRNA, miR-27a, by directly binding to its promoter region (42). miR-27a binds to the 3′ UTR of the *Il10* mRNA and thus mediates its decay (27). We demonstrated that miR-27a activity is required for restraint of IL-10 protein expression during infection. In the absence of HIF-1α, infection with H. capsulatum led to a decrease in the expression of miR-27a and thus to elevated quantities of *Il10* transcript and IL-10 protein. This level of reduction in miR-27a expression is biologically relevant in other models (27, 42). In our model, this defect in miR-27a expression in *Lyz2cre Hif1α^fl/fl^* macrophages is not observed in uninfected cells, indicating that the stressor of H. capsulatum infection is required to serve as a second signal to drive the downregulation of miR-27a in HIF-1α-deficient macrophages. While we do not exclude the possibility that HIF-1α regulates miR-27a via direct activity at the promoter region of this miRNA as reported in other models (42), our data indicate that the key mechanism of HIF-1α-mediated regulation of miR-27a expression during H. capsulatum infection occurs via restraint of fumarate accumulation. Infection is required for accrual of HIF-1α protein (12). The failure of infection (and, thus, HIF-1α activity) to perturb the net quantity of miR-27a in wild-type cells suggests that the question of whether HIF-1α directly regulates miR-27a may be of less importance to our model than the identity of the negative regulators responsible for the decline in miR-27a expression in HIF-1α-deficient macrophages.

HIF-1α mediates the comprehensive reprogramming of macrophages in response to infection-related cues via rewiring of cellular metabolism. Stimulation with LPS or β-glucan induces a Warburg phenotype characterized by elevated glycolysis and diminished mitochondrial respiration. In the absence of HIF-1α, H. capsulatum fails to induce lactate production, as quantified by extracellular lactate and the extracellular acidification rate. However, no additional defects in glycolysis, such as decreased glucose uptake, hexokinase, and GAPDH activity, or pyruvate production was observed in HIF-1α-deficient macrophages. Our data support previous work (28) indicating that H. capsulatum does not stimulate a classical Warburg phenotype, as intracellular infection of both HIF-1α-sufficient and -deficient macrophages led to an increased rate of mitochondrial respiration. HIF-1α-deficiency enhanced this phenotype, suggesting that while H. capsulatum infection results in a net elevation of mitochondrial respiration, HIF-1α promotes some degree of restraint of oxidative phosphorylation (OXPHOS). The diverse cues provided by intracellular infection promote complex immunometabolic reprogramming that may not be fully recapitulated by classical tools of immune stimulation such as LPS and β-glucan. The assortment of pattern-associated molecular patterns displayed by pathogens trigger discrete yet often synergistic signaling pathways within the host macrophage. Additionally, stimulation with LPS or β-glucan does not encapsulate the dynamic interplay between a living intracellular pathogen and its host, including nutrient competition. The diversity encompassed by host-pathogen interactions likely fuels, and is supported by, the variable nature of the macrophage immunometabolic profile.

Warburg metabolism of LPS-activated macrophages is supported by two critical HIF-1α-dependent disruptions in the TCA cycle characterized by decreased isocitrate dehydrogenase and succinate dehydrogenase activity (17, 43, 44). These breakpoints permit the accumulation of metabolic intermediates that serve as substrates for the formation of antimicrobial products as well as signals for posttranslational protein modification and epigenetic rewiring of the macrophage, with the net effect being the proinflammatory activation of the cell. Conversely, our data indicate that, in the absence of HIF-1α, a deleterious break in the TCA cycle at fumarate hydratase (FH) promotes accumulation of fumarate during H. capsulatum infection. Thus, breaks in this metabolic cycle appear to depend on the stimulus. Here, decreased *Fh* expression and FH activity occurs independently of infection, yet accumulation of fumarate requires both H. capsulatum infection and the absence of HIF-1α. One possible explanation is that the limited quantity of FH in HIF-1α-deficient BMDMs may be adequate for conversion of fumarate to malate during resting metabolism. Infection, however, stimulates increased succinate dehydrogenase activity, which may overwhelm the enzymatic capacity of FH, leading to accumulation of fumarate.

The anti-inflammatory effects of metabolites such as pyruvate and fumarate or their pharmacological derivatives, especially dimethyl fumarate, have been widely reported in the literature (30–32, 45–52). Fumarate has been associated with increased IL-10 (30–32). However, the mechanism(s) by which these metabolites promote IL-10 expression are unknown. TCA cycle metabolites, including fumarate, modify the epigenome via competitive inhibition of α-ketoglutarate-dependent DNA and histone demethylases (33, 34). Excessive fumarate inhibits Tet-mediated DNA demethylation of a CpG island in the promoter region of the miR-200 cluster, resulting in the decreased expression of these anti-metastatic miRNAs (35). Loss of Tet2 expression is associated with elevated plasma IL-10 (36). In macrophages, fumarate inhibits KDM5-mediated histone demethylation (9). Here, we report that accumulation of fumarate in the absence of HIF-1α correlates with decreased activity of the α-ketoglutarate-dependent KDM5 subfamily of histone demethylases. Inhibition of fumarate production increased total KDM5 activity and treatment with exogenous fumarate was shown to oppose α-ketoglutarate-mediated restraint of IL-10 protein production. These data implicate decreased KDM5 histone demethylation activity due to accumulation of fumarate as a driver of IL-10 protein production in the absence of HIF-1α. However, neither infection nor the absence of HIF-1α was sufficient to change the global quantity of H3K4me3, one of the targets of KDM5 histone demethylation. While these data do not eliminate the possibility that KDM5 demethylation is relevant to regulation of miR-27a expression, they do strongly indicate that alternative targets of fumarate should be explored. One possible hypothesis is that excessive fumarate may inhibit DNA demethylation by one or more members of the α-ketoglutarate-dependent Tet family. DNA methylation at a CG-rich region within the promoter of the miR-23a-27a-24-2 cluster has been shown to regulate expression of these miRNAs (53). A key question is whether decreased Tet family activity due to fumarate accumulation has a direct or indirect effect on the expression of either miR-27a or *Il10* mRNA.

This study employed BMDMs as an analog for the cells responsible for excessive IL-10 production in our mouse model of infection, namely, lung F4/80^+^ CD11b^+^ CD11c^−^ macrophages isolated from *Lyz2cre Hif1α^fl/fl^* mice (12). We previously demonstrated that infection of BMDMs with H. capsulatum induces accrual of HIF-1α protein and transcription of *Hif1a* and its downstream targets (12). Here, we confirm that *ex vivo* F4/80^+^ lung cells from *Lyz2cre Hif1α^fl/fl^* mice express a higher quantity of *Il10* mRNA but less miR-27a than cells from wild-type controls. Additionally, we demonstrate that *in vivo* treatment with the TCA cycle inhibitors UK5099 and DMM resulted in decreased lung IL-10 (but not TNF-α) and decreased fungal burden. Under nonhypoxic conditions, human peripheral blood monocyte-derived macrophages accrue HIF-1α protein in the nucleus in response to H. capsulatum infection (28). Treatment of infected human monocyte-derived macrophages with a pharmaceutical stabilizer of HIF-1α reduced H. capsulatum growth (28). As proof of concept, fumarate has been shown to inhibit KDM5 activity in opposition to α-ketoglutarate in human monocytes in a model of immune training (9) and human embryonic kidney cells (10).

Direct or indirect pharmacological suppression of HIF-1α may increase the susceptibility of patients to H. capsulatum infection and disease. Corticosteroid and anti-TNF-α therapy are known risk factors for development of histoplasmosis due to direct suppression of the inflammatory response to infection. Emerging evidence suggests that such therapies may also indirectly suppress HIF-1α expression or activity. In a model of Aspergillus fumigatus infection, treatment with the corticosteroid triamcinolone decreased HIF-1α protein in nuclear extracts prepared from the whole lung compared to untreated controls (14). TNF-α promotes accrual of HIF-1α in macrophages under normoxia (54), suggesting that anti-TNF-α therapy may limit one of the cues responsible for induction of HIF-1α. Recently, the diversity of the HIF-1α regulome has made this transcription factor a popular target for pharmacological intervention (55–58). The broad regulome of this essential transcription factor, however, also portends the diversity of deleterious effects that may result from the suppression of HIF-1α activity. Here, we provide evidence that diminished HIF-1α activity critically disrupts the proinflammatory function of macrophages during intracellular infection via comprehensive rewiring of the TCA cycle.

## MATERIALS AND METHODS

### Mice.

*Lyz2cre/cre* (B6.129P2*-Lyz2^tm1^*^(^*^cre^*^)^*^Ifo^*/J The Jackson Laboratory) and *Lyz2cre/cre Hif1α^fl/fl^* mice were housed in isolator cages and maintained by the Department of Laboratory Animal Medicine, University of Cincinnati, accredited by the Association for Assessment and Accreditation of Laboratory Animal Care. For the *Ldha* experiments (Fig. 2E and F), *Lyz2cre/cre Ldha^fl/fl^* mice [B6(Cg)-*Ldha^tm1c^*^(^*^EUCOMM^*^)^*^Wtsi^*/DatsJ; The Jackson Laboratory] and *Lyz2cre/cre* (B6.129P2-*Lyz2^tm1^*^(^*^cre^*^)^*^Ifo^*/J; The Jackson Laboratory) control mice were housed in isolator cages and maintained by the Division of Veterinary Services at Cincinnati Children’s Hospital Medical Center (CCHMC), accredited by the Association for Assessment and Accreditation of Laboratory Animal Care. All animal experiments were performed in accordance with the Animal Welfare Act guidelines of the National Institutes of Health, and all protocols were approved by the Institutional Animal Care and Use Committees of the University of Cincinnati or CCHMC.

### Preparation of H. capsulatum and infection of mice.

H. capsulatum strain G217B and yeast cells of the same strain that express GFP were grown for 72 h at 37°C as described elsewhere (59, 60). To infect mice, 6- to 8-week-old animals were inoculated intranasally with 2 × 10^6^ yeasts in 60 μl Hanks balanced salt solution (HBSS) (HyClone). Macrophages were infected with five yeasts per host cell for the indicated times.

### Generation of BMDMs.

Bone marrow was isolated from tibiae and femurs of 6- to 10-week-old mice by flushing with HBSS. Cells were dispensed into tissue culture flasks at a density of 10^6^ cells/ml of complete RPMI 1640 supplemented with 10% fetal bovine serum, 0.1% gentamicin sulfate, 5 mM 2-mercaptoethanol, and 10 ng/ml recombinant mouse granulocyte-macrophage colony-stimulating factor (GM-CSF; Miltenyi Biotec). Flasks were incubated at 37°C in 5% CO_2_. Fresh cell culture medium was added at day 3. At day 7, nonadherent cells were removed, and BMDMs were scraped from the flask following digestion with trypsin. BMDMs were collected, washed with phosphate-buffered saline (PBS), and dispensed into culture dishes in complete RPMI without GM-CSF. Cells were seeded at 2 × 10^5^ macrophages per well in a standard 96-well tissue culture plate. Cells were allowed to adhere to the plate overnight at 37°C in 5% CO_2_ before treatment and/or infection. Incubation of BMDMs following treatment and/or infection occurred in a standard 5% CO_2_ incubator at 37°C, with near-atmospheric oxygen conditions (i.e., not hypoxia).

### RNA isolation, cDNA synthesis, and quantitative real-time RT-PCR.

For analysis of miRNAs, total RNA from BMDMs or bead-selected F4/80^+^ lung macrophages was isolated using the *mir*Vana miRNA isolation kit (Thermo Fisher). The TaqMan microRNA reverse transcription kit (Thermo Fisher) was used to generate cDNA. Quantitative real-time PCR analysis was performed using TaqMan Universal Master Mix II, no uracil-*N*-glycoslyase (UNG), and primers (Thermo Fisher) specific for hsa-miR-27a-3p (assay ID 000408), hsa-miR-98-5p (assay ID 000577), mmu-miR-106a (assay ID 002459), miR-142-3p (assay ID 000464), hsa-let-7c (assay ID 000379), and the endogenous control sno234 (assay ID 001234). The conditions for amplification were 50°C for 2 min and 95°C for 10 min, followed by 40 cycles of 95°C for 15 s and 60°C for 1 min.

The PureLink RNA minikit (Thermo Fisher) was used for RNA isolation applications without concurrent miRNA analysis. Oligo(dT)-primed cDNA was prepared using the reverse transcriptase system (Promega). Quantitative real-time PCR analysis was performed on the Applied Biosystems 7500 fast real-time PCR system (Thermo Fisher) using TaqMan fast universal PCR master mix (no UNG) and TaqMan gene expression assay primers (Thermo Fisher) specific for *Il10* (mm00439616_m1), *Sdha* (mm01352366_m1), *Fh1* (mm01321349_m1), and the endogenous control, *Hprt* (mm00446968_m1). The conditions for amplification were 50°C for 2 min and 95°C for 10 min, followed by 40 cycles of 95°C for 15 s and 60°C for 1 min.

For the glucose metabolism PCR array, mRNA was isolated from BMDMs at 24 h postinfection using the PureLink RNA minikit. Single-stranded cDNA was synthesized from 1 μg of total RNA using the RT^2^ first-strand kit (Qiagen). Gene expression was examined using the Qiagen mouse glucose metabolism RT^2^ Profiler PCR array (PAMM-006Z) with RT^2^ SYBR green ROX qPCR master mix via the Applied Biosystems 7500 fast real-time PCR instrument (Thermo Fisher Scientific). Gene expression was calculated according to the relative quantification method (2^−ΔΔ^*^CT^*). Samples were analyzed in triplicate and normalized based on the expression of internal housekeeping genes (*Actb*, *Gapdh*, *Hprt1*, and *Rplp0*).

### Analysis of miR-27a and *Il10*.

Stability of the *Il10* transcript was evaluated via quantitative real-time PCR at 0, 60, 120, and 180 min after treatment with an 8 μM concentration of the transcriptional inhibitor actinomycin D. Gene expression was calculated according to the relative quantification method (2^−ΔΔ^*^CT^*). The samples were analyzed in triplicate and normalized based on the expression of the 18S rRNA (assay ID hs99999901_s1; Thermo Fisher). The half-life was calculated according to a two-parameter exponential decay curve using SigmaPlot 12.5. has-miR-27a-3p *mir*Vana miRNA mimic (catalog no. 4464066, assay ID MC10939; Thermo Fisher), inhibitor (catalog no. 4464084, assay ID MH10939; Thermo Fisher), or scrambled RNA control or target site blocker (Qiagen) was transfected into BMDMs using Lipofectamine RNAiMAX (Thermo Fisher). The miRCURY LNA miRNA Power Target site blocker (Qiagen), mmu-miR-27a-3p (3′ CGCCUUGAAUCGGUGACACUU 5′), was designed based on the known target site of miR-27a on the 3′ UTR of the *Il10* mRNA (27). BMDMs were isolated from tissue culture flasks at day 7 of differentiation (as described above) and seeded at 2 × 10^5^ macrophages per well in a standard 96-well tissue culture plate. Cells were allowed to adhere to the plate for 3 to 4 h at 37°C in 5% CO_2_ before treatment. The miR-27a mimic, inhibitor, target site blockers, or scrambled RNA control was diluted to 50× of the final concentration and then mixed 1:1 with transfection reagent in Opti-MEM (Thermo Fisher). The transfection suspension was incubated for 5 min at room temperature and then added to BMDMs to a final concentration of 30 nM. Cells were incubated for 18 h at 37°C in 5% CO_2_ before transfection medium was replaced with fresh complete RPMI. Isolation of total RNA for real-time RT-PCR occurred at 24 h postinfection, while the supernatant was collected at 48 h postinfection for quantification of IL-10 and TNF-α cytokines via mouse DuoSet ELISA (BioTechne).

### Characterization of metabolic phenotype.

Extracellular lactate (Cayman Chemical), and intracellular pyruvate, succinate and fumarate were quantified via colorimetric kit (BioVision) according to the manufacturer’s directions. The relative enzymatic activities of hexokinases 1 to 4, glucose-6-phosphate dehydrogenase (G6PDH), succinate dehydrogenase, and fumarate hydratase were determined by colorimetric kit (BioVision) according to the manufacturer’s directions. Expression of glucose transporter (Glut1) on infected cells was measured by flow cytometry following surface staining of BMDMs with Alexa Fluor 647-conjugated anti-Glut1 antibody clone EPR3915 (Abcam). Infected BMDMs were identified based on the detection of green fluorescent protein-positive (GFP^+^) H. capsulatum. Relative glucose uptake was measured via incubation of BMDMs with the fluorescent d-glucose analog 2-[N-(7-nitrobenz-2-oxa-1,3-diazol-4-yl)amino]-2-deoxy-d-glucose (2-NBDG; Thermo Fisher). Data were acquired using a BD Accuri C6 cytometer (BD Biosciences) and analyzed using FCS Express 6 (De Novo Software).

For the glycolytic and mitochondrial respiration rate assays, BMDMs were seeded at 4 × 10^4^ cells in 180 μl complete RPMI per well in Seahorse XF 96-well culture microplates (Agilent) and rested overnight at 37°C in 5% CO_2_. On the day of the assay, the cell culture medium was replaced with 180 μl Seahorse XF Dulbecco’s modified Eagle medium (DMEM) containing 10 mM glucose, 2 mM l-glutamine, and 1 mM sodium pyruvate followed by a 1-h incubation at 37°C in ambient CO_2_. The basal glycolytic proton efflux rate (glycoPER) was measured with a Seahorse XF glycolytic rate assay kit using a Seahorse XF96e analyzer (Agilent). Glycolytic proton efflux rate was calculated via subtraction of nonglycolytic acidification (as measured after 2-deoxy-d-glucose [2-DG] treatment) from the total proton efflux rate. Basal mitochondrial respiration was measured via Seahorse XF Cell Mito stress test kit using a Seahorse XF96e analyzer (Agilent). The basal oxygen consumption rate (OCR) was calculated via subtraction of nonmitochondrial oxygen consumption (as measured after rotenone and antimycin A treatment) from the total oxygen consumption rate.

### LC-MS analysis.

Samples were homogenized and extracted using a mixture of 3:3:2 (vol/vol/vol) acetonitrile (Honeywell Burdick & Jackson)-isopropanol-water. Cells were subjected to cold solvent extraction, scraped from the dish, vortexed, and then centrifuged at room temperature to pellet cell debris. Samples were resuspended in 450 ml 50:50 acetonitrile-H_2_O, vortexed, and centrifuged. The pellet was discarded, and the supernatant was dried in a SpeedVac. Dried samples were resuspended in 80:20 mobile phase A to mobile phase B (MPA-MPB) and subjected to LC-MS analysis.

Separation was accomplished by hydrophilic interaction liquid chromatography (HILIC) using a 2.0- by 150-mm (5-μm particle size, 100-Å pore size) PEEK Shodex HILICpak VN-50 column, on a Vanquish Flex quaternary ultrahigh-performance liquid chromatography (UHPLC) system (Thermo Fisher). Mobile phase A consisted of 25 mM ammonium bicarbonate (Thermo Fisher) in LC-MS-grade water (Alfa Aesar), pH 6.0. Mobile phase B consisted of acetonitrile with a gradient of 20% B (from 0 to 2 min), 28% B at 10 min, and 95% B at 10.1 min, held for 2 min, and then returned to 20% B at 12.1 min at a flow rate of 100 μl/min. The column temperature was set at 40°C.

MS analyses was performed on an Orbitrap Fusion Lumos Tribrid mass spectrometer (Thermo Fisher) interfaced with an heated-electrospray ionization (H-ESI) electrospray source in negative-polarity mode. Data were acquired on the Orbitrap using MS1 profiling at a resolution of 30,000, mass range of 114 to 700 *m/z*, automatic gain control (AGC) of 1e6, and ion injection time (IT) of 50 ms. Additional settings were as follows: wide quad isolation, true; mass range, normal; radio frequency (RF), 25%; sheath gas, auxiliary gas, and sweep gas, 45, 5, and 1 arbitrary units, respectively; ion transfer tube temperature, 325°C; vaporizer temperature, 350°C; and spray voltage, 2,700 V. Data were analyzed by Tracefinder 4.1 using an external calibration curve constructed against known standard retention time profiles with a mass accuracy of <5 ppm.

### TCA cycle inhibition.

BMDMs were subjected to concurrent H. capsulatum infection and treatment with UK5099 (Cayman Chemical), an inhibitor of pyruvate transport into the mitochondrion, or dimethyl malonate (DMM, Sigma-Aldrich), a competitive inhibitor of succinate dehydrogenase. Cytokine gene expression and extracellular cytokines were quantified at 24 or 48 h posttreatment, respectively, as described above. The impact of TCA cycle inhibition on H. capsulatum viability was measured in yeasts grown in culture medium or host BMDMs after 24 h of either direct or indirect exposure (respectively) to UK5099 and DMM. Yeasts from culture medium or lysed from host BMDMs were resuspended in sterile HBSS, serially diluted, and plated on Mycosel (Becton Dickinson Co.) agar plates containing 5% sheep blood and 5% glucose. Plates were incubated at 30°C for 7 days. The limit of detection was 10^2^ CFU. Flow cytometry was used to quantify 7-amino-actinomycin D (7-AAD^+^) dead cells versus 7-AAD^−^ live BMDMs after 48 h of concurrent treatment with inhibitor and H. capsulatum. The impact of TCA cycle inhibition on basal mitochondrial respiration (OCR) was measured with the Seahorse Cell Mito stress test as described above.

ON-TARGETplus mouse siRNA *Sdha* SMARTpool (catalog no. L-046818-01; Horizon Discovery) and siRNA *Fh1* SMARTpool (catalog no. D-001810-10; Horizon Discovery) or scrambled RNA control were transfected into BMDMs using TransIT-TKO transfection reagent (Mirus Bio) to a final concentration of 20 nM according to the manufacturer’s instructions. Cells were incubated for 18 h at 37°C in 5% CO_2_ before transfection medium was replaced with fresh complete RPMI. Total RNA was isolated using the *mir*Vana miRNA isolation kit (Thermo Fisher) at 24 h postinfection. Supernatant was collected at 48 h postinfection for quantification of IL-10 and TNF-α cytokines via mouse DuoSet ELISA (BioTechne).

### Treatment with exogenous TCA metabolites.

BMDMs were treated with 10 nM dimethyl fumarate or 1 mM dimethyl α-ketoglutarate (Santa Cruz) at the time of H. capsulatum infection. Mitochondrial respiration was analyzed by Seahorse assay at 24 h postinfection and extracellular cytokines were quantified by ELISA at 48 h postinfection, as described above.

### Quantification of KDM5 activity.

The fluorometric KDM5/JARID activity quantification assay kit (Abcam) was used to measure demethylation of histone H3K4 substrate following incubation with 2 μg of nuclear extracts collected from BMDMs via nuclear extraction kit (Abcam) after 24 h of concurrent H. capsulatum infection and treatment with 10 μM DMM. The colorimetric histone H3 trimethylated Lys4 ELISA (Active Motif) was used to measure the total quantity of H3K4me3 in acid-extracted proteins collected from BMDMs after at 24 h postinfection. Proteins were extracted in 0.4 M HCl, followed by neutralization with 1 M sodium phosphate (dibasic, pH 12.5) supplemented with 2.5 mM dithiothreitol (DTT) and 10 mM phenylmethylsulfonyl fluoride (PMSF).

### Isolation of F4/80^+^ lung macrophages.

At 3 days postinfection, lungs were homogenized with a gentleMACS dissociator (Miltenyi Biotec) in 5 ml complete RPMI with 2 mg/ml collagenase D and 40 U DNase I (Roche) for 30 min at 37°C. Erythrocytes were removed using ammonium-chloride-potassium (ACK) lysis buffer (150 mM NH_4_Cl, 10 mM KHCO_3_, 0.1 mM Na_2_EDTA). Cells were filtered through a 40-mm nylon mesh, washed, and counted. Anti-F4/80 MicroBeads were used for positive selection of F4/80^+^ cells (Miltenyi Biotec).

### *In vivo* treatment with UK5099 and DMM.

Mice were treated by the intraperitoneal route with 12 mg/kg UK5099, 600 mg/kg DMM, or vehicle (DMSO) 1 day prior to and for 2 days following H. capsulatum infection. Mice were sacrificed 3 days after infection. Lungs and spleens were homogenized in complete RPMI, serially diluted in PBS, and plated onto Mycosel agar plates containing 5% sheep blood and 5% glucose. Plates were incubated at 30°C for 7 days. The limit of detection was 10^2^ CFU.

### Statistical analyses.

For the mouse glucose metabolism RT^2^ Profiler PCR array, data were analyzed using the GeneGlobe Data Analysis Center (Qiagen). Student’s *t* tests (parametric, unpaired, two-sample equal variance, two-tailed distribution) were used to compare gene expression between infected *Lyz2cre* and *Lyz2cre Hif1α^fl/fl^* BMDMs. For the mass spectrometry data, Student’s *t* tests (parametric, unpaired, two-sample equal variance, two-tailed distribution) were used to compare the quantities of each metabolite between infected *Lyz2cre* and *Lyz2cre Hif1α^fl/fl^* BMDMs. For all other assays, one-way or two-way analysis of variance (ANOVA) were used to determine differences between groups, with Student-Newman-Keuls *post hoc* testing. Data were determined to be significantly different when the *P* value was <0.05.
